# Population-based incidence trends of oropharyngeal and oral cavity cancers by sex among the poorest and underprivileged populations

**DOI:** 10.1186/1471-2407-14-316

**Published:** 2014-05-05

**Authors:** Ajit Auluck, Blake Byron Walker, Greg Hislop, Scott A Lear, Nadine Schuurman, Miriam Rosin

**Affiliations:** 1 Biomedical Physiology and Kinesiology, Simon Fraser University, Burnaby, Canada; 2 Department of Geography, Simon Fraser University, Burnaby, BC, Canada; 3 Faculty of Health Sciences, Simon Fraser University, Burnaby, Canada; 4 Division of Cardiology, Providence Health Care, St. Paul’s Hospital, Vancouver, Canada; 5 Cancer Control Research Department, BC Cancer Agency, Research Centre, 675 W. 10th Ave, 3rd Floor, Room 119, V5Z1L3 Vancouver, B.C, Canada

**Keywords:** HPV infection, Oral cavity cancer, Oropharyngeal cancer, Incidence, Socioeconomic deprivation

## Abstract

**Background:**

Oral cancer is an important health issue, with changing incidence in many countries. Oropharyngeal cancer (OPC, in tonsil and oropharygeal areas) is increasing, while oral cavity cancer (OCC, other sites in the mouth) is decreasing. There is the need to identify high risk groups and communities for further study and intervention. The objective of this study was to determine how the incidence of OPC and OCC varied by neighbourhood socioeconomic status (SES) in British Columbia (BC), including the magnitude of any inequalities and temporal trends.

**Methods:**

ICDO-3 codes were used to identify OPC and OCC cases in the BC Cancer Registry from 1981–2010. Cases were categorized by postal codes into SES quintiles (q1-q5) using VANDIX, which is a census-based, multivariate weighted index based on neighbourhood average household income, housing tenure, educational attainment, employment and family structure. Age-standardized incidence rates were determined for OPC and OCC by sex and SES quintiles and temporal trends were then examined.

**Results:**

Incidence rates are increasing in both men and women for OPC, and decreasing in men and increasing in women for OCC. This change is not linear or proportionate between different SES quintiles, for there is a sharp and dramatic increase in incidence according to the deprivation status of the neighbourhood. The highest incidence rates in men for both OPC and OCC were observed in the most deprived SES quintile (q5), at 1.7 times and 2.2 times higher, respectively, than men in the least deprived quintile (q1). For OPC, the age-adjusted incidence rates significantly increased in all SES quintiles with the highest increase observed in the most deprived quintile (q5). Likewise, the highest incidence rates for both OPC and OCC in women were observed in the most deprived SES quintile (q5), at 2.1 times and 1.8 times higher, respectively, than women in the least deprived quintile (q1).

**Conclusion:**

We report on SES disparities in oral cancer, emphasizing the need for community-based interventions that address access to medical care and the distribution of educational and health promotion resources among the most SES deprived communities in British Columbia.

## Background

Although health equity is a fundamental goal of many health care systems, it is well documented that inequalities in health outcomes exist both within and between countries. For example, adult mortality rates are twice as high in blacks as in whites in the United States [1] and almost three times as high in unskilled workers as in professionals in the United Kingdom [2]. An important reason for these differences relates to socioeconomic inequities because people residing in poorer neighbourhoods have higher prevalence of high risk behaviours such as smoking and alcohol and less access to health care services.

Disparities in incidence have been observed at multiple scales, varying between global regions [3-5], within countries [6,7], and between neighbourhoods [8]. It is known that socioeconomic inequalities persist in cancer incidence [9] but little recognition has been given to the effects of socioeconomic status (SES) on the risk of developing oral cancers [10], a cancer showing significant change in trajectory worldwide [11-14]. A study from Scotland suggested that the risk for oral cancer is higher among people living in deprived neighbourhoods (OR = 4.66), a finding mainly attributed to higher rates of smoking (OR = 15.53) [6]. Another study from Canada suggested that SES status affects incidence of oral cancer, with higher rates reported among people with lower median income, less than 8^th^ grade education and visiting dentists less than once a year [15]. Although several studies have shown associations between SES and oral cancer risk, none have shown an independent effect of SES on risk for developing oral cancer. Conway et al. [16] conducted a systematic review and meta-analysis exploring the relationship between socioeconomic inequalities and oral cancer risk. Their research suggested that in comparison to populations with higher SES, the risk of developing oral cancer was 1.85 times higher with lower educational attainment, 1.84 times higher with low occupational social class and 2.41 times higher with lower income. Further, they suggested that lower SES was significantly associated with increased oral cancer risk in high and lower income countries, which remained after adjusting for potential behavioural confounders. However, after controlling for age, sex, smoking, and alcohol consumption, SES was no longer a significant variable [6,7,17]. Therefore, it is important to ascertain the risk of oral cancers according to SES status.

In our previous research in British Columbia (BC), we found that the incidence is increasing among both men and women for oropharyngeal cancers (OPC, in tonsil and oropharygeal areas), and decreasing among men for oral cavity cancer (OCC, other sites in the mouth) [18]. These observed differences were attributed to differences in the aetiology of oral cancers at these different sites [4,12]; however, it is also important to determine how SES, which may influence the prevalence of risk behaviours (such as alcohol consumption, smoking, and oro-sexual practices) and access to health care [16,19], is related to differences in incidence rates. Studies on SES disparities in oral cancer research are emerging from the European Union [20], Scotland [6,7], California (US) [19], and Canada [15,17,21]; however, these studies have their own limitations, such as a lack of site-specific [6,15,20] and sex-specific [6,15,21] data and small sample sizes [6,15]. A recent paper from California highlighted the importance of reporting population-based trends of oral cancers by site, SES and sex [19]. The objective of our paper is to analyse the relationship between neighbourhood SES status (using a composite index with multiple socioeconomic features including income, housing, education, family demographics and employment obtained from both census data and local health surveys) [22,23] and incidence for both OPC and OCC stratified by sex, using the population-based cancer registry in BC.

## Methods

### Study population

This study was approved by the research ethics boards at the BC Cancer Agency (certificate number HO8-00839) and Simon Fraser University (2012-s-0348). Our study was conducted in the province of BC, Canada, which had a population of 4,113,487 persons in 2006. In BC, cancer is a reportable disease to the population-based BC cancer registry (BCCR). BCCR, established in 1969, maintains a high quality database, consistently recording more than 85% of all cancer cases in the province, and has well-established linkages with BC Vital Statistics database to capture death data. The quality of data is found to be acceptable for inclusion in the North American Association of Central Cancer Registries (NAACCR) and International Agency for Research on Cancer (IARC).

Cases were identified from the BCCR for the period from 1981 to 2010, with selection based on histological diagnosis of invasive squamous cell carcinoma in the oral cavity or oropharynx, as defined by the International Classifications of Diseases in Oncology, 3rd edition (ICDO-3). Morphology codes for selected cases included if they were suggestive of invasive characteristics: 80003, 80103, 80203, 80213, 80323, 80333, 80503, 80513, 80523, 80703, 80713, 80723, 80733, 80743, 80753, 80763, 80833, 80943 and 81233. Site codes were then used for etiological clustering of cases into OPC and OCC excluding tumours at external lips (COO-C001), salivary glands (C079, C080), nasopharynx (C119) and hypopharynx (C139) and as described in our earlier papers [18,24], since these cancers are associated with other etiological factors. This resulted in identifying 2059 and 4319 cases of primary OPC and OCC, respectively, for a total of 6378 cases that were included in the analysis. Registry data were collected on cancer characteristics including anatomic site (location of the tumour in the head and neck region), histology (morphology of the tumour), date of diagnosis (when the tumour diagnosis was first made), and tumour stage (extent and severity of the cancer based on tumour size, lymph node involvement and evidence of metastasis); and patient demographics including name, age at the time of tumour diagnosis, and sex or gender. Since ethnicity and place of birth are not recorded in the BCCR, South Asian (SA) and Chinese cases were identified from the selected cases using previously generated ethnic surname lists [25,26]. When surnames of cases were found to match the ethnic surname list, these names were then manually verified by SA and Chinese researchers.

### Neighbourhood socioeconomic status

Residential neighbourhood socioeconomic deprivation was calculated for each of the 2006 Census Dissemination Blocks (DB) in BC (N = 55,505). The mean population of a DB is 79 residents, providing sub-neighbourhood scale socioeconomic data. The Vancouver Area Neighbourhood Deprivation Index (VANDIX) [22] score was calculated for each 2006 Census Dissemination Area (DA) (N = 6,900). VANDIX is a census-based, composite weighted index based on neighbourhood average household income, percentage of population living at one address for the previous five years, percentage of population with post-secondary and without secondary school education, workforce participation rate, and percentage of single-parent households. Variable weights were derived from local surveys of provincial medical health officers [22,23]. The resulting VANDIX value for each DA was then assigned to the appropriate DBs (one DA containing an average of 8 DBs) and the DBs were categorised into deprivation quintiles (q1-q5), based on the VANDIX values across the entire province.

The socioeconomic deprivation quintile q1 represented the least deprived neighbourhoods and q5 represented the most deprived neighbourhoods. Using Geographic Information Systems (GIS), neighbourhood socioeconomic deprivation was linked to individual patients from the BCCR by joining the 6-digit postal codes of a patient’s residence to the VANDIX deprivation index and the resulting neighbourhood deprivation quintiles were used for the subsequent incidence analysis.

### Statistical analysis

Differences in demographic and clinicopathologic characteristics between OPC and OCC were tested for significance using Student’s *t*-test, Pearson’s chi-square test and one-way ANOVA [27]. OPC and OCC age-adjusted incidence ratios (AAIR) and age-specific incidence rates (ASIR) with 95% confidence intervals (CI) were calculated separately by neighbourhood deprivation quintiles [28]. The AAIR were standardized to the 1991 BC general population. In order to examine temporal trends in incidence, AAIR were then calculated in 5-year intervals for the total time period 1981 to 2010. The annual percent change (APC) in incidence rates was then calculated by fitting a least squares regression line to the natural logarithm of the rates, using the calendar year as the regression variable, rejecting the null hypothesis that APC equals 0 if the resulting p-value was <0.05. The results were presented separately for men and women. All calculations were done using SPSS (Statistical Package for the Social Sciences) version 20.

## Results

The demographic and clinical characteristics of the study population are described in Table 1. There were 2059 and 4319 cases of OPC and OCC, respectively. The majority of both OPC and OCC were in men, with 1512 (73.4%) and 547 (26.6%) of OPC cases in men and women, respectively, and 2692 (62.3%) and 1627 (37.7%) of OCC cases in men and women, respectively. Mean age at diagnosis for men was significantly lower than for women for both OPC (P < 0.001) and OCC (P < 0.001). Greater proportions of OPC cases were diagnosed in more recent years for men than women (P = 0.004), whereas greater proportions of OCC cases were diagnosed in more recent years for women than men (P < 0.001). Significantly more OCC cases were found in South Asian and Chinese women than men (P = 0.00 l). Finally, greater proportions of both OPC and OCC cases were diagnosed with later stage disease in men than women (P < 0.001).

**Table 1:** Demographic and clinical characteristics of the study population by sex

**Characteristic**	**OPC**	**OCC**	**P**	**OPC**	**OCC**	**P**	**P’**	**P”**
	**Men**			**Women**				
**Mean age at tumour diagnosis**	60.5 ± 10.9	63.6 ± 13.2	<0.001*	63.5 ± 12.3	67.1 ± 14.8	<0.001*	<0.001*	<0.001*
**Age**								
*<50 years*	287 (19.0%)	413 (15.3%)	<0.001*	76 (13.9%)	213 (13.1%)	<0.001*	<0.001*	<0.001*
*51-60 years*	492 (32.5%)	633 (23.5%)		159 (29.1%)	294 (18.1%)			
*61-70 years*	447 (29.6%)	782 (29.0%)		138 (25.2%)	406 (25.0%)			
*71 years & above*	286 (18.9%)	864 (32.1%)		174 (31.8%)	714 (43.9%)			
**Year of tumour diagnosis**								
*1981-85*	99 (6.5%)	416 (15.5%)	<0.001*	55 (10.1%)	217 (13.3%)	0.008*	0.004*	<0.001*
*1986-90*	160 (10.6%)	481 (17.9%)		71 (13.0%)	229 (14.1%)			
*1991-95*	201 (13.3%)	491 (18.2%)		84 (15.4%)	287 (17.6%)			
*1996-2000*	245 (16.2%)	471 (17.5%)		88 (16.1%)	301 (18.5%)			
*2001-05*	393 (26.0%)	446 (16.6%)		137 (25.0%)	310 (19.1%)			
*2006-10*	414 (27.4%)	387 (14.4%)		112 (20.5%)	283 (17.4%)			
**Ethnicity**								
*South Asian*	42 (2.8%)	97 (3.6%)	0.37	20 (3.7%)	77 (4.7%)	0.02*	0.04*	0.001*
*Chinese*	51 (3.4%)	85 (3.2%)		10 (1.8%)	83 (5.1%)			
*General population*	1419 (93.8%)	2510 (93.2%)		517 (94.5%)	1467 (90.2%)			
**Stage at diagnosis**								
*Early*	211 (14.0%)	907 (33.7%)		124 (22.7%)	615 (37.8%)	<0.001*	<0.001*	<0.001*
*Late*	1245 (82.3%)	1138 (42.3%)		392 (71.7%)	598 (36.8%)			
*Unknown*	56 (3.7%)	647 (24.0%)		31 (5.7%)	414 (25.4%)			

P value is based on independent T-test value for mean age at diagnosis.

P value is based on P value based on Pearson’s chi -square test for age, year of diagnosis, ethnicity, stage at diagnosis.

P’ based on difference between OPC men and women.

P” based on difference between OCC men and women.

*Indicates that P value is significant (level of significance <0.05).

OPC- oropharyngeal cancers; OCC- oral cavity cancers.

Sample sizes for men with OPC in each socioeconomic deprivation quintile were: q1 (n = 266, 17.6%), q2 (n = 252, 16.6%), q3 (n = 237, 15.6%), q4 (n = 306, 20.2%), and q5 (n = 451, 29.8%); while for OCC they were q1 (n = 412, 15.3%), q2 (n = 437, 16.2%), q3 (n = 427, 15.8%), q4 (n = 509, 18.9%), and q5 (n = 907, 33.6%). Sample sizes for women with OPC in each quintile were: q1 (n = 49, 17.2%), q2 (n = 54, 18.9%), q3 (n = 43, 15.1%), q4 (n = 59, 20.7%), and q5 (n = 80, 28.1%); while for OCC they were q1 (n = 166, 16.1%), q2 (n = 170, 16.5%), q3 (n = 192, 18.6%), q4 (n = 240, 23.2%), and q5 (n = 265, 25.7%). Mean ages at diagnosis were calculated for OPC and OCC by sex and SES deprivation quintiles but no statistically significant differences were found (data not shown).

### Men

#### Age-adjusted incidence rates (AAIR) for OPC and OCC by SES quintile

**Figure 1:**
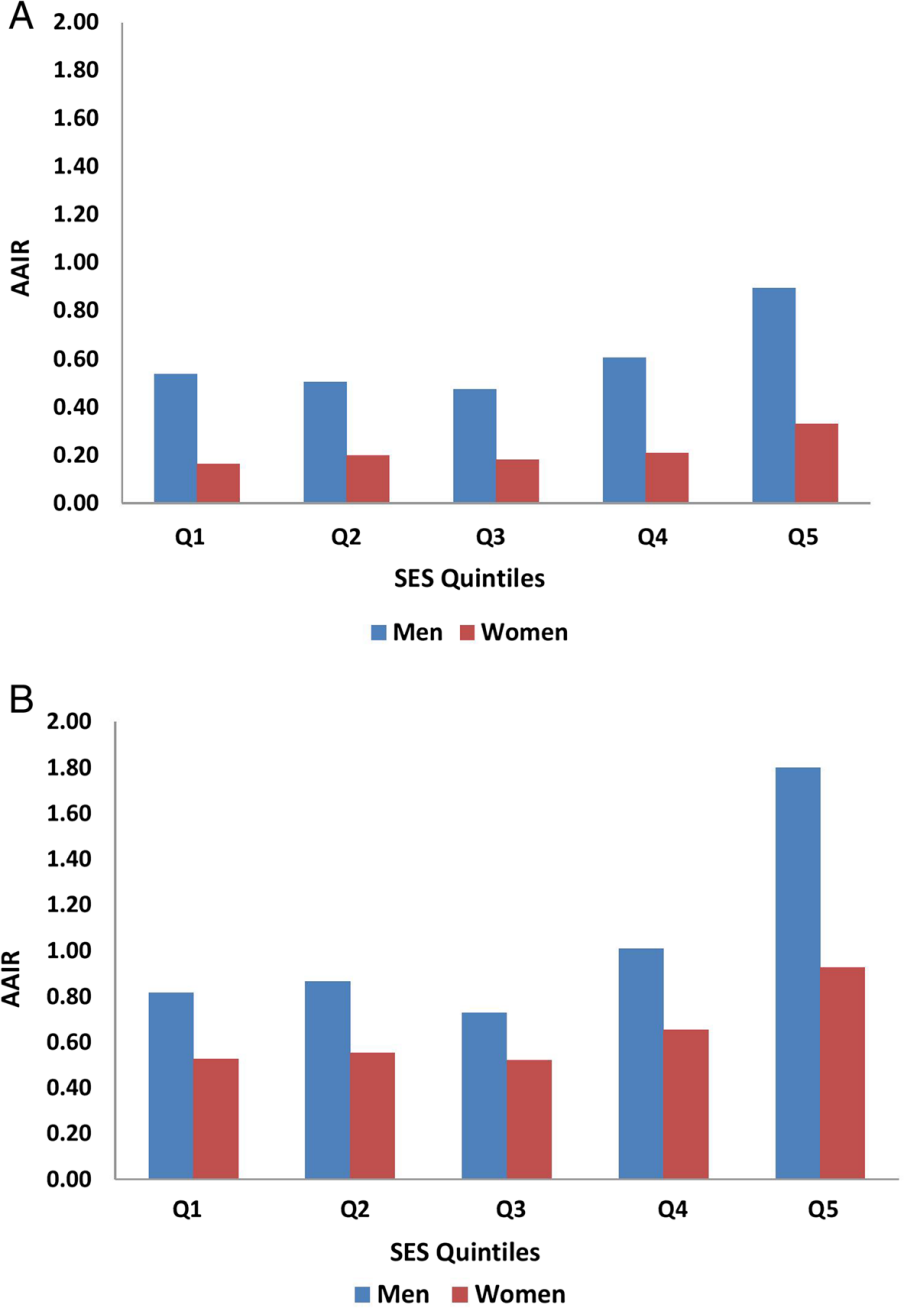
**AAIR (age-adjusted incidence rates) from 1981–2010 by gender and SES (socioeconomic status) quintile: (A) for OPC (oropharyngeal cancers) and (B) for OCC (oral cavity cancers).** Age-adjusted incidence rates for oropharyngeal and oral cavity cancers.

The AAIR for OPC and OCC are shown by SES deprivation quintiles and gender in Figure 1 for the total study period from 1981 to 2010. For OPC in men, AAIR for quintiles 1 (least deprived) to 5 (most deprived) were: 0.54 (95% CI, 0.28 – 0.80); 0.51 (95% CI, 0.26 – 0.75); 0.47 (95% CI, 0.24 – 0.70); 0.61 (95% CI, 0.34 – 0.87); and 0.89 (95% CI, 0.58 – 2.12), respectively. For OCC in men, AAIR for the deprivation quintiles 1 to 5 were: 0.82 (95% CI, 0.49 – 1.14); 0.87 (95% CI, 0.53 – 1.20); 0.73 (95% CI, 0.56 – 1.21); 1.01 (95% CI, 0.65 – 1.37); and 1.80 (95% CI, 1.33 – 2.26). The highest incidence rates in men for both OPC and OCC were observed in the most deprived quintile (q5), at 1.7 times and 2.2 times higher than men in the least deprived quintile (q1) for OPC and OCC, respectively. Of interest, AAIR for both OPC and OCC were similar in the less deprived quintiles (q1-3).

#### Temporal trends in AAIR for OPC and OCC by SES quintile

**Figure 2:**
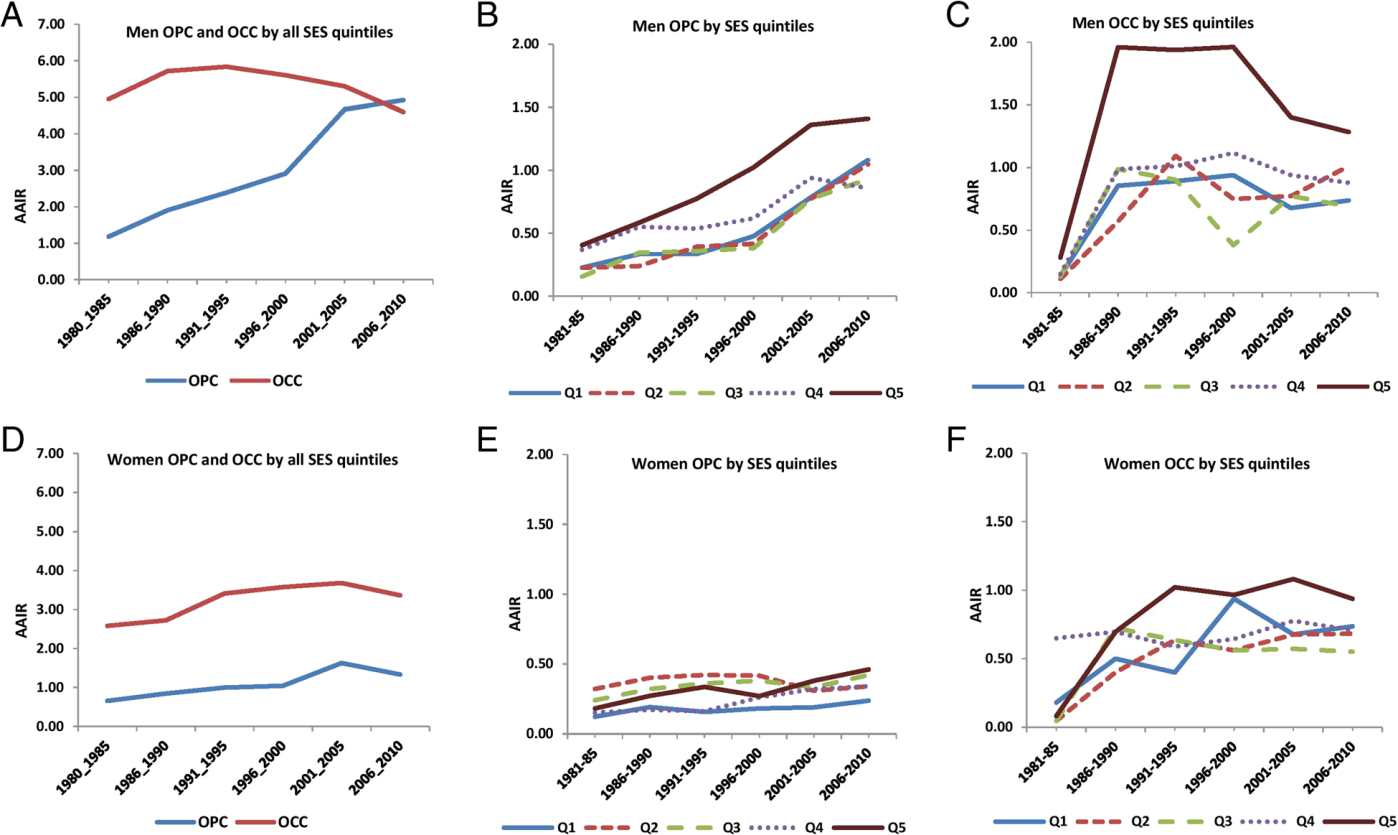
**Temporal trends in AAIR (age-adjusted incidence rates) from 1981-2010 for OPC (oropharyngeal cancers) and OCC (oral cavity cancers) by SES (socioeconomic status) quintiles and sex.** Q1 is the least deprived quintile while Q5 is most deprived quintile. **(A)** OPC and OCC for men, for all quintiles together. **(B)** OPC in men, by quintile. **(C)** OCC in men, by quintile. **(D)** OPC and OCC for women, for all quintiles together. **(E)** OPC in women, by quintile. **(F)** OCC by women, by quintile.

Figure 2A shows AAIR for men in all SES deprivation quintiles combined from 1981 to 2010, in which the AAIR for OPC significantly increased from 1.18 (95% CI, 0.81-1.55) to 4.92 (95% CI, 4.22-5.63), with an annual percent change (APC) of 0.97 (P <0.001). However, the AAIR for OCC decreased non-significantly from 4.95 (95% CI, 4.16-5.74) to 4.60 (95% CI, 3.83-5.37), with an APC of - 0.36 (P = 0.47). Of note, the AAIR for OPC surpassed OCC in 2006–2010. These AAIR trends were also observed in the different SES deprivation quintiles (Figure 2B and C). For OPC, the AAIR significantly increased in all quintiles with the highest increase observed in the most deprived quintile (q5). The APCs were 0.96 (P = 0.001), 0.95 (P = 0.001), 0.90 (P = 0.004), 0.87 (P = 0.006), and 0.98 (P < 0.001) for SES deprivation quintiles q1 to q5, respectively. For OCC, the APC initially increased and then levelled off in all quintiles, with a decline apparent in 2001–2005 and later; these results being non-significant. The APCs were 0.38 (P = 0.24), 0.53 (P = 0.10), 0.22 (P = 0.34), 0.37 (P =0.19) and 0.29 (P = 0.34) for SES deprivation quintiles 1 to 5, respectively. Of interest, the largest decline in OCC rates was seen in q5.

**Figure 3:**
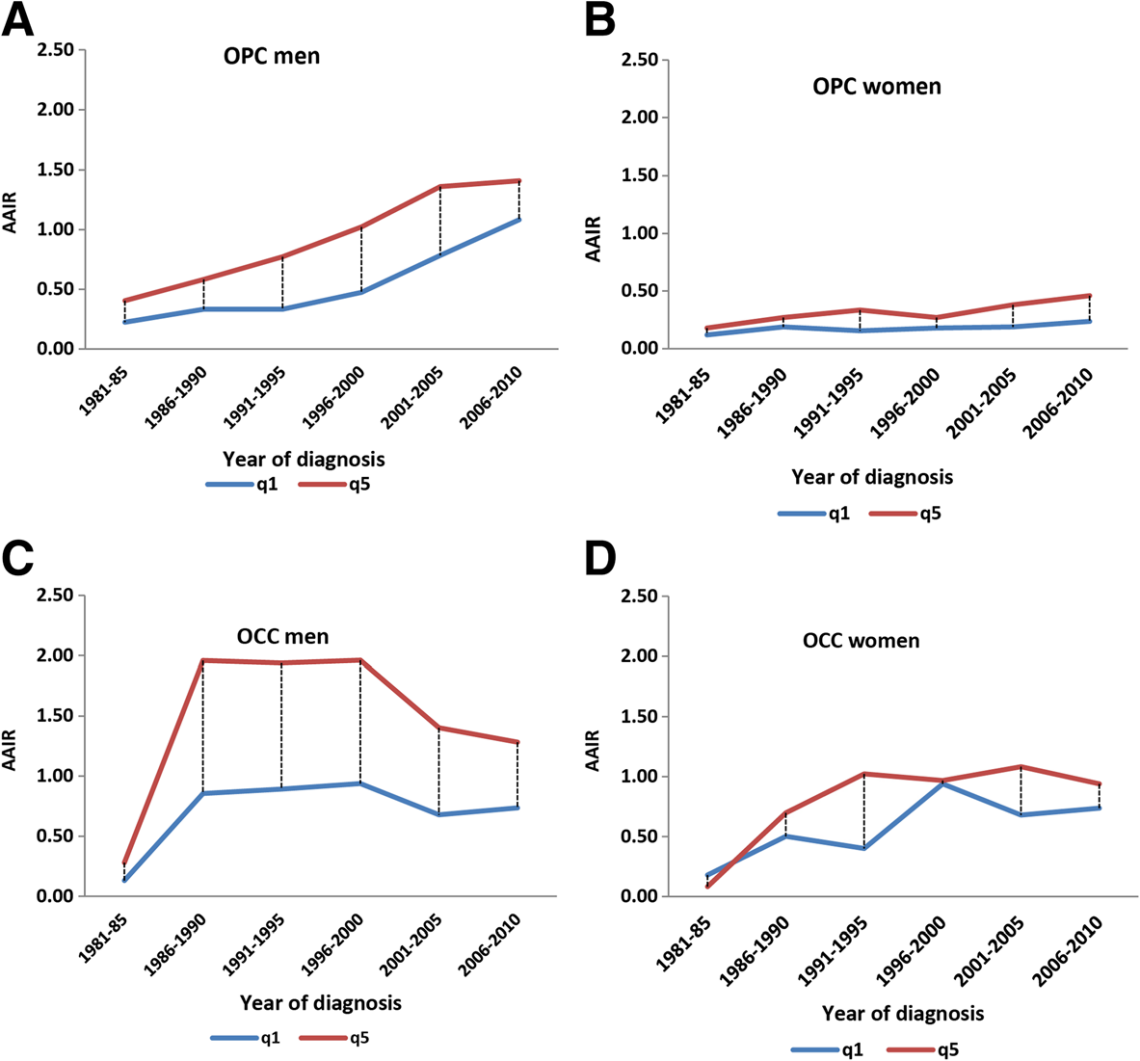
**Temporal trends in disparities gap or inequalities in AAIR (age-adjusted incidence rates) from 1981-2010 for OPC (oropharyngeal cancers) and OCC (oral cavity cancers).** Comparisons are between the least (q1) and most deprived (q5) and by sex. **(A)** OPC in men. **(B)** OPC in women. **(C)** OCC in women. **(D)** OCC in women.

Figures 3A and C show change over time in inequalities in the AAIR for men in comparisons of the least (q1) and most deprived (q5) quintiles. For OPC, first the disparities widened and then narrowed in 2006–2010. The narrowing inequalities between 2001–05 and 2006–2010 were associated with a significant increase in incidence rates in q1 from 0.78 to 1.08 with only a marginal increase in q5 from 1.36 to 1.41. For OCC, there was a minimal disparity during 1981–85 but this widened considerably between 1986–2000. Thereafter, disparities narrowed because of lower incidence rates in both q1 and q5. Overall, disparities were highest for OCC among men.

#### Age-specific incidence rate (ASIR) in the most deprived quintile (q5)

**Figure 4:**
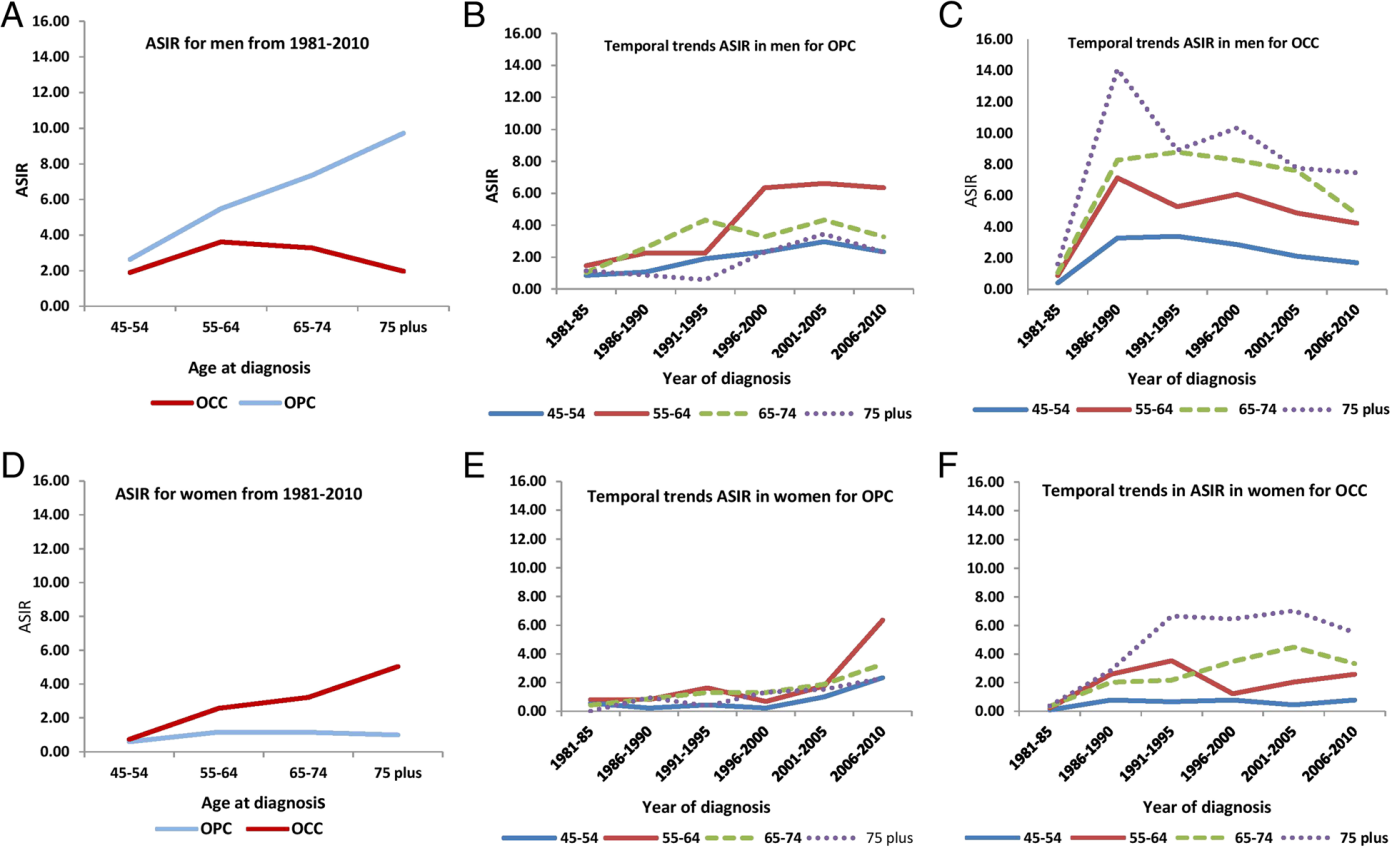
**ASIR (age-standardized incidence rates) for OPC (oropharyngeal cancers) and OCC (oral cavity cancers) in the lowest SES (socioeconomic status) quintile (q5).** Comparisons are made by sex. For men: **(A)** By age, over the total study period, comparing OPC and OCC. **(B)** Temporal trends by age for OPC. **(C)** Temporal trends by age for OCC. For women: **(D)** By age over the total study period. **(E)** Temporal trends by age for OPC. **(F)** Temporal trends by age for OCC.

We then calculated ASIRs for OPC and OCC in men in the most deprived quintile (q5), as these groups showed the highest incidence rates for both OPC and OCC (Figure 4A-C). As expected, OCC incidence rates increased with increasing age and the maximum burden of disease was observed among those age 75 years and older. The highest increase in OPC incidence was observed in the age group 55–64 years. For OPC, ASIRs in q5 were: 1.89 (95% CI, 1.41-2.83) for 45–54 years; 3.62 (95% CI, 2.43-4.19) for 55–64 years; 3.28 (95% CI, 1.52-4.23) for 65–74 years; and 1.97 (95% CI, 0.43-4.97) for 75 years and older (Figure 4A). For OCC, ASIRs in q5 were: 2.64 (95% CI, 2.12–3.84) for 45–54 years; 5.49 (95% CI, 3.91-6.20) for 55–64 years; 7.36 (95% CI, 4.12-9.61) for 65–74 years; and 9.73 (95% CI, 3.17-17.82) for 75 years and older (Figure 4A).

Temporal trends in ASIRs for OPC and OCC in men in the most deprived quintile (q5) are shown in Figure 4B and C. The highest ASIRs for both OPC and OCC were seen in recent years (2006–2010). The ages of highest ASIR differed for OPC and OCC, being ages 55–64 years and 75 years and older, respectively.

### Women

#### Age-adjusted incidence rates (AAIR) for OPC and OCC by SES quintile

Similar to men, the highest incidence rates in women for both OPC and OCC were observed in the most deprived quintile (q5) (Figure 1). For OPC in women, AAIRs for SES quintiles 1 to 5 were: 0.16 (95% CI, 0.04 – 0.29); 0.20 (95% CI, 0.05 – 0.36); 0.18 (95% CI, 0.04 – 0.32); 0.21 (95% CI, 0.05 – 0.36); and 0.33 (95% CI, 0.14 – 0.52), respectively. For OCC in women, AAIRs for quintiles 1 to 5 were: 0.53 (95% CI, 0.26 – 0.79); 0.55 (95% CI, 0.30 – 0.81); 0.52 (95% CI, 0.27 – 0.77); 0.66 (95% CI, 0.36 – 0.95); and 0.93 (95% CI, 0.59 – 1.26), respectively. Women in the most deprived quintile (q5) were at highest risk for both OPC and OCC, being 2.1 times higher for OPC and 1.8 times higher for OCC than women in the least deprived quintile (q1). Of interest, the AAIRs for both OPC and OCC in women were similar for quintiles q1 to q3, with an increase observed in quintile 4 and a further increase in quintile 5.

#### Temporal trends in AAIR for OPC and OCC by SES quintile

Figure 2D shows AAIRs for women in all SES deprivation quintiles combined from 1981 to 2010, in which the AAIR for OPC significantly increased from 0.65 (95% CI, 0.39-0.92) to 1.33 (95% CI, 0.94-1.72), with an annual percent change (APC) of 0.91 (P =0.009). Unlike men, the rates of OCC among women are still increasing from 2.58 (95% CI, 2.01-3.15) to 3.69 (95% CI, 3.02-4.35), with an APC of 0.58 (P = 0.05). The observed crossover in trajectories for OPC incidence versus OCC seen in men (Figure 2A) is not apparent in women.

These AAIR trends were also observed in the different SES deprivation quintiles (Figure 2E and F). For OPC, the AAIR significantly increased in all quintiles except q2 with the highest increase observed in the most deprived quintiles (q4 and q5). The APCs were 0.82 (P = 0.04), -0.09 (P = 0.71), 0.82 (P = 0.04), 0.95 (P = 0.005), and 0.90 (P < 0.01) for SES deprivation quintiles q1 to q5, respectively. For OCC, the APC increased in all quintiles but no significant increase was observed in any quintile. The APCs were 0.38 (P = 0.24), 0.29 (P = 0.34), 0.22 (P = 0.34), 0.37 (P =0.19) and 0.53 (P = 0.10) for SES deprivation quintiles 1 to 5, respectively. When changes in the most deprived quintile (q5) are examined, the increase is much steeper for men compared to women. The incidence of OCC for men in q5 showed a peak in 1986–1990, then a plateau followed by a decreased incidence around 1996–2000. However, the incidence rates of OCC for women gradually increased and approached those observed in men in 2006–10.

Figure 3B and D show changes in inequalities in the AAIR for women by comparing the least (q1) and most deprived (q5) quintiles. For OPC, there was little difference between these 2 quintiles although the gap in incidence appears to have widened in recent years, with higher incidence rates in q5. For OCC, the disparity between q1 and q5 is generally larger than that observed with OPC (except for the 1996–2000 time period). In both instances, the disparity between q1 and q5 was higher for men than women for both OPC and OCC.

#### Age-specific incidence rates (ASIR) in the most deprived quintile (q5)

We then calculated ASIRs for OPC and OCC in women in the most deprived quintile (q5), as these groups showed the highest incidence rates for both OPC and OCC (Figure 4D-F). As expected, OCC incidence rates increased with increasing age and the maximum burden of disease was observed among those age 75 years and older. The highest increase in OPC incidence was observed two decades earlier, in the age group 55–64 years. For OPC, ASIRs in q5 were: 0.57 (95% CI, 0.28-0.92) for 45–54 years; 1.16 (95% CI, 0.88-1.49) for 55–64 years; 1.13 (95% CI, 0.82-1.53) for 65–74 years; and 0.98 (95% CI, 0.43-1.96) for 75 years and older (Figure 4E). For OCC, ASIRs in q5 were: 0.72 (95% CI, 0.42–1.04) for 45–54 years; 2.56 (95% CI, 1.91-3.29) for 55–64 years; 3.21 (95% CI, 2.12-4.91) for 65–74 years; and 5.04 (95% CI, 3.17-11.82) for 75 years and older (Figure 4F).

Temporal trends in ASIRs for OPC and OCC in women in the most deprived quintile (q5) are shown in Figure 4E and F. The highest ASIRs for OPC and OCC were seen in recent years (2006–2010) in the age group of 75 years and older. Of note, differences in temporal trends in ASIRs for OPC and OCC were more pronounced among men than women.

## Discussion

Our findings contribute to the growing body of global literature showing the increasing incidence of OPC [11-14,29,30] and provide sociodemographic information on those at high risk of both OPC and OCC in BC. To our knowledge, this is the first study in Canada examining temporal trends in incidence for OPC and OCC by sex and neighbourhood socioeconomic deprivation status using a population-based cancer registry. Our analysis revealed important findings that among men the incidence of OPC has now surpassed the incidence rates of OCC, and this increase in the incidence rates is seen across all neighbourhood SES quintiles. Although OPC incidence rates increased over time in all quintiles, the highest incidence was observed among men living in the most deprived neighbourhoods (q5); more specifically, among men ages 55–64 years. Among women, the incidence rates of both OCC and OPC are significantly increasing, with highest rates observed in the most deprived quintile (q5). Interestingly, the temporal trends in incidence rates for OPC and OCC differed in men and women, with widening of inequalities between the least (q1) and most deprived (q5) quintiles in 2006–10 as compared to 1981–85. These findings add to current research relating to associations between socioeconomic deprivation and health outcomes even in a country such as Canada with a universal health care system.

These observed gender differences in the temporal trends in the incidence rates for OPC and OCC can partially be explained by differences in smoking rates. In BC, smoking rates are declining in both sexes. Among men, smoking rates have decreased from 51% to 17.9% from 1965 to 2007. Decline in rates among women began later and was less dramatic; the rates have decreased from 38% to 11.1%, respectively [31]. More dramatic and earlier decreases in smoking rates of men may partially explain the differences in OCC incidence rates over time – the sharp decline in rates of OCC among men that has occurred concurrent to rates of OCC among women that are still increasing. For q5, this change means that incidence frequencies for women are approaching those seen in men in this quintile. The observed increase in incidence of OPC among both men and women is most likely related to the increased prevalence of human papilloma virus (HPV), which is associated with these tumours. Nicholas et al. [32] recently reported from Ontario that during 1993–99 to 2006–2011 the prevalence of HPV in oropharyngeal cancers increased from 25% to 62%; this is likely also the case in BC. Therefore, future research about the risk behaviours of OPC and OCC should target populations in the least deprived neighbourhoods, paying specific attention to patterns in the men and women.

Our findings are consistent with other Canadian studies from Ottawa [15] and from data of Canadian Cancer Registry [21] which showed that the highest head and neck cancer incidence was observed among populations with the lowest incomes. However, these studies did not provide sex-specific rates and their assessment of SES status was restricted to only material deprivation indices such as income. Many case–control and epidemiological studies suggest that prevalence of oral HPV infection is greater among men than women, which supports the observation that HPV-related OPC is predominantly seen in men [33-35]. In our study OPC rates have increased dramatically among men; women show similar but less dramatic trends. Our study shows the importance of reporting sex-specific rates when examining impact of factors such as SES status.

In contrast to the results of a recent study from Canada [21], which reported no significant change in rates of OCC over time, we found significant differences by gender, with a declining incidence of OCC in men but increasing in women. Hwang et al. [21] reported that lowest income quintiles had higher rates of OPC (66%) and OCC (48%) but that there was no significant narrowing of the gap between the highest and lowest quintiles. However, our study suggests a widening gap in incidence in BC in recent years as compared to 1981–5 for both OCC and OPC in both genders. Therefore, performing sex-based analysis and using VANDIX as a measure to determine SES status provided helpful insight and should be considered in future studies.

Living in wealthier neighbourhoods has been associated with better self-rated health resulting from better lifestyles and healthier choices [36]. People living in more deprived neighbourhoods have stresses associated with poverty and unemployment which can itself lead to cancer development [37] and studies have shown an increasing gradient of cancer with increasing socioeconomic deprivation [16,38]. Further, people may be more inclined to smoke tobacco as a coping mechanism to deal with the stresses of poverty [39], which further increases their cancer risk. They may also have less access to social services. Increased burden of OPC and OCC among men in the most deprived neighbourhoods in our study may be explained in part by higher rates of smoking, less exercise, and poorer diets (e.g., eating less fruit) [38]. In BC, men have higher rates of smoking prevalence than women and these rates may be even higher in poorer neighbourhoods, which supports the observed higher rates of oral cancers [40]. Higher prevalence of risky behaviours and oral cancer burden was previously reported from Vancouver’s Downtown Eastside, one of the most socioeconomically deprived neighbourhoods in Canada [41]. Hence, new developing technologies such as optical screening devices for detection of OCC need to include the poor and underprivileged communities to obtain maximum benefit. Our findings also emphasize the need to continuously develop community outreach programmes and target the most deprived and vulnerable populations for oral cancer screening and prevention.

Although there is a need to develop targeted prevention approaches for the deprived neighbourhoods it’s important to also address the broader social determinants of health and address the underlying causes of inequitable distribution of wealth and resources which influences the lifestyle and risk behaviours increasing their risk for developing oral cancers [42]. Poor clients need to be empowered and encouraged to participate in health promotion and prevention services for reducing smoking and alcohol consumption, encouraged to adopt a healthy lifestyle and efforts should be made to improve their access to health care facilities [2,43]. We need an integrated approach and political action for framing the social and health polices to tackle the root causes of disadvantage.

### Strengths and limitations

Our study benefits from several strengths. Firstly, we used data from the population-based BCCR over three decades to determine the incidence of OPC and OCC. This allowed us to have a sufficiently large sample size to present data separately for OPC and OCC by sex and to focus our analysis upon men who have a higher oral cancer burden. Secondly, we only selected biopsy-confirmed OCC and OPC cases, which eliminated potential errors of over-inclusion of cases, a problem encountered in individual surveys where biopsy results are often not included. Thirdly, we used a composite peer-reviewed index (VANDIX) [22] to determine the SES deprivation status of neighbourhoods which included weighted proportions of multiple socioeconomic features obtained from both census data and local health surveys; it has been used in other studies as well [44]. And fourthly, by using the registry which contained 6-digit postal codes for each case’s place of residence, we were able to assign neighbourhood deprivation status to each case. This permitted us to identify the most SES deprived neighbourhoods and observed that these places also suffered from the highest burdens of OPC and OCC.

Our study has limitations similar to other registry-based studies. BCCR does not record parameters to determine individual SES, nor does it record a patient’s HPV status or risk behaviours such as smoking or alcohol consumption. By using neighbourhood deprivation as a proxy for individual SES, actual differences in incidence by socioeconomic status may be obscured. However, inferring a patient’s individual SES deprivation based upon neighbourhood deprivation level is the only currently reliable method available to determine trends by socioeconomic status in the absence of such data in cancer registries or other databases. Another limitation is that neighbourhood deprivation cannot identify individuals who do not have a permanent residence address. Exclusion of such marginalized groups may lead to an underestimation of disease burden. Although we provide evidence of a higher burden of disease in the most deprived neighbourhoods we do not explore the pathways and processes associated with SES and its relationship to OCC and OPC incidence. Further research is needed using population-based surveys and qualitative studies to understand the risk behaviours of populations in the most deprived neighbourhoods. Finally, there may be some inherent error in our use of the VANDIX index for it contained variables from the 2006 census which may change over time and not accurately reflect the neighbourhoods over the total study period.

## Conclusion

Our study provides evidence that the burden of oral cancers is highest in the most SES deprived populations in BC. Interestingly, this increase in the incidence rates of oral cancers is not linear or proportionate between different quintiles, but there is a sharp and dramatic increase in the incidence rates according to the deprivation status of the neighbourhood. Given a global increase in incidence of OPC and its association with HPV, this analysis of a population-based cancer registry and neighbourhood socioeconomic deprivation provides novel findings of increased disease burden of OPC in the most deprived neighbourhoods. This is an important evidence for informing targeted neighbourhood-based interventions (such as oral cancer screening, awareness, prevention and HPV vaccination) and facilitating a more focussed strategy on oral cancer prevention in BC. It also contributes to the growing body of global literature about epidemiological trends of oral cavity and oropharyngeal cancers, which is important for making policies and strategies for global oral cancer control and prevention.

As a next step, we will use geographic information systems (GIS) to identify clusters of cases in these high-risk neighbourhoods and relate these clusters to catchment areas for cancer clinics. This research is important because BC has a comprehensive oral cancer prevention programme, and the results of our research will help in the development of neighbourhood-based targeted approaches for health promotion, harm reduction, oral cancer screening, and prevention programmes for the most vulnerable and highest-risk population groups.

## Abbreviations

BC: British Columbia; OCC: Oral cavity cancer; OPC: Oropharyngeal cancer; BCCR: British Columbia cancer registry; VANDIX: Vancouver area neighbourhood deprivation index; AAIR: Age-adjusted incidence ratios; ASR: Age-specific incidence rates; APC: Annual percentage change; CI: Confidence interval; GIS: Geographical information systems; HPV: Human papilloma virus; SA: South Asian.

## Competing interests

The authors declare that they have no competing interests.

## Authors’ contributions

AA – was involved in data acquisition, data coding, data analysis, data interpretation and writing of this manuscript. BBW – was involved in data coding and writing of this manuscript. GH – contributed in data interpretation, provided feedback on scientific content and revising the manuscript. SL – contributed in data presentation, providing feedback on scientific content and revising the manuscript. NS – contributed in creating VANDIX, data coding and revising the manuscript. MR – was involved in conceptual design of this project, guiding data analysis, interpretation, preparing and revising the manuscript. All authors provided consent to the final version of this manuscript.

## Pre-publication history

The pre-publication history for this paper can be accessed here:

http://www.biomedcentral.com/1471-2407/14/316/prepub
